# Periodicity as a Character of Disease

**Published:** 1860-12

**Authors:** Richard Hughes

**Affiliations:** M. R. C. S., L. R. C. P. Ed., Surgeon to the Brighton Orthopædic Hospital


### PERIODICITY AS A CHARACTER OF DISEASE.

BY RICHARD HUGHES, M. R. C. S., L. R. C. P. ED., SURGEON TO THE BRIGHTON ORTHOPÆDIC HOSPITAL.

There is one disease—ague—of the phenomena of which periodicity is an invariable characteristic. There are other affections—the neuralgia and nervous headaches—which very frequently assume a regular intermittent character. And, occasionally, in the course of various diseases a tendency to a periodical type is manifested.

Quinine and arsenic are specifics in ague. They are hardly less valuable in neuralgia and nervous headaches. And when, in works on medicine, we meet with the remark that sometimes the symptoms seem to come on periodically, it is generally coupled with the statement that in these cases quinine will be found of the utmost value.

What is the *rationale* of the facts?

The cause of the regular periodic recurrence of the paroxysms of intermittent fever, is discussed by Dr. Watson, in the first volume of his Lectures on Medicine, p. 758. After passing in review the various theories that have been advanced to account for it, he leaves the subject as one altogether uncertain. Cullen's hypothesis as to the influence of diurnal habit he thinks the nearest approach to the truth; but admits that this will in no way account for the tertian and quartan types of ague. In this state of uncertainty, the peculiar poison—malaria—which causes intermittent fever has been generally regarded as the source of its periodical character. It is supposed to act in the way of a ferment in the blood: and the zymotic process set up by it is supposed to have its regular development and decline, the paroxysms being its effect on the nervous system when the process attains its acme. When other affections manifest a periodic type, it is supposed that their subjects have either had ague, or been exposed to malarious influences. On the other hand, quinine and arsenic are considered to be antidotes to the malarious poison, and thus to counteract its periodic influence in all affections which tend to assume that type. But I think I shall be borne out by general experience when I assert that periodic phenomena are manifested in many a case of neuralgia or other disease, when the hypothesis of malarious influence is altogether shut out. This theory, therefore, in itself *a priori* most improvable, must be at once rejected.

If, then, the periodicity of ague does not depend upon the peculiar poison which occasions it, does it depend upon the peculiar portion of the organism which that poison affects? This is universally acknowledged to be the nervous system—the sympathetic.

Let me place one after another some of the principal phenoma of the cold stage of ague, with those produced by galvanization of the sympathetic in the neck.

In the cold stage of ague "the patient feels chilly; the blood deserts the superficial capillaries; he grows pale, his features shrink, his skin is rendered dry and rough, his respiration is quick and anxious; his pulse frequent sometimes, but feeble; all the secretions are usually diminished; may make water often, though generally he voids but little, and it is pale and aqueous; his bowels are confined, and his tongue is dry and white."— *Watson, op. cit., p.* 735.

Let Dr. Brown-Sequard now tell us the result of his galvanizing the sympathetic in the neck. "The pupils are dilated; the eyelids wide open; the globes protruding. Blood deserts the superficial vessels; secretion is diminished or checked; temperature and all vital functions lowered."

It is clear from the above that that the essential phenomena (diminished afflux of blood to the surface, check of secretion, and lowering of temperature) of the cold stage of intermittent fever may be produced artificially by excitation of the sympathetic nerve, and are dependent upon the contraction of the blood-vessels thereby occasioned. The hot stage is no less explicable as the natural reaction of the vessels to even beyond their normal calibre, and finds its precise analogue in the phenomena which results from section of the sympathetic in the neck.

It would appear from the above that the sympathetic system is that part of the organism specially affected by the malarious poison, and that the phenomena of ague depend on a periodic excitation of it by the poison, followed by an equally immoderate reaction in the opposite direction, which latter at length settles down to the equilibrium of health. And upon the hypothesis the beneficial influence of quinine and arsenic in this disease receives its perfect *rationale*. For these are well known as (to use Dr. Handfield Jones' term) "toners of the vaso-motor nerves"—i. e., of the sympathetic, and their tonic influence thus exerted will obviously render it capable of resisting the morbid irritation of the malarious poison—a view of which is confirmed by the well known fact that quinine is no less valuable as a preventive than as a curative agent against this malady.

But will the fact of the sympathetic system being the seat of the phenomena of ague help us to explain the periodic character of these phenomena? I think it will. Let us consider the actions of the heart and the uterus, two muscular organs supplied mainly or entirely by this nerve.

What is the cause of the rhythm of the heart? It cannot be the stimulus of the blood, or of anything else within the body: for a frog's heart will continue its regular systole and diastole for some time after its removal from the thorax. The only motive agency then left to it is that of the sympathetic ganglia, which are imbedded in its substance. To these nervous centres we are therefore shut in our inquiry as to the heart's action; but the influence of these centres is neither continuous nor occassional, but rhythmical—i. e., periodic.

The uterus resembles the heart in its also possessing numerous sympathetic ganglia imbedded in its muscular walls, and in entire independence of the cerebro-spinal system in its movements. Is there anything here periodic? We immediately think of the regular monthly recurrence of the phenomena of menstruation, and of the act of parturition, which (as first shown by Dr. Tyler Smith in the pages of *The Lancet*) is determined by the menstrual periods, being normally due on the 21st day after the commencement of the last menstruation.*

From the marked periodicity apparent in the action of these two organs, exclusively animated by sympathetic ganglia we seem justified in the conclusion that a periodical evolution of nerve-force is characteristic of this division of the nervous system; and if this be the case, we cannot be surprised that it should give its peculiar character to phenomena resulting from the effect of a poison upon itself. Thus the periodic character of the paroxysms of ague is reduced under the mere general law of periodic character of phenomena, morbid or natural, over which the sympathetic system presides, dependent upon the endowment of this system itself.

It follows that periodic phenomena appearing in the course of any other diseases must be attributed to an involvement of the sympathetic system in their morbid irritation; and thus the beneficial effects of quinine and arsenic in all such cases fall under the general law of their toning influence upon that system. I may instance the most common affection which

*This theory should be more widely carried out in obstetric practice. I have been delighted at the exactitude with which it has enabled me to predict even the day of delivery. The advantage of such a pre-knowledge, whether to patient, or practitioner, is immense.

inclines to a periodic type—nervous headache. Dr. Symods, in his admirable Gulstonian Lectures upon this subject, shows by many arguments that the vascular nerves (i. e., sympathetic) of the brain and skull are the seat of pain in this affection, and in common with general experience, regards quinine and arsenic as the sheet-anchor in its treatment.

I venture to admit that the following original conclusions are established by the preceding considerations:—1. That ague s an affection of the sympathetic system. 2. That its periodical character is dependent upon a periodicity impressed upon the sympathetic system, and manifested in all the phenomena, morbid and natural, over which it presides. 3. That the *modus operandi* of quinine and arsenic in ague and other periodical affections is the toning influence exerted by them on the sympathetic nerve. The doctrine also of the dependence of the heart's movements on its embedded ganglia, and of the menstrual periodicity as the determining cause of parturition, receive no slight corroboration; and our instinctive recourse to quinine and arsenic, whenever periodic symptoms manifest themselves, receives alike its *rationale* and its establishment.

